# Manufacturing clinical‐grade human induced pluripotent stem cell‐derived beta cells for diabetes treatment

**DOI:** 10.1111/cpr.13232

**Published:** 2022-04-26

**Authors:** Lay Shuen Tan, Juin Ting Chen, Lillian Yuxian Lim, Adrian Kee Keong Teo

**Affiliations:** ^1^ Stem Cells and Diabetes Laboratory Institute of Molecular and Cell Biology, A*STAR Singapore Singapore; ^2^ Department of Biochemistry, Yong Loo Lin School of Medicine National University of Singapore Singapore Singapore; ^3^ Department of Medicine, Yong Loo Lin School of Medicine National University of Singapore Singapore Singapore; ^4^ Precision Medicine Translational Research Programme (TRP), Yong Loo Lin School of Medicine National University of Singapore Singapore Singapore

## Abstract

The unlimited proliferative capacity of human pluripotent stem cells (hPSCs) fortifies it as one of the most attractive sources for cell therapy application in diabetes. In the past two decades, vast research efforts have been invested in developing strategies to differentiate hPSCs into clinically suitable insulin‐producing endocrine cells or functional beta cells (β cells). With the end goal being clinical translation, it is critical for hPSCs and insulin‐producing β cells to be derived, handled, stored, maintained and expanded with clinical compliance. This review focuses on the key processes and guidelines for clinical translation of human induced pluripotent stem cell (hiPSC)‐derived β cells for diabetes cell therapy. Here, we discuss the (1) key considerations of manufacturing clinical‐grade hiPSCs, (2) scale‐up and differentiation of clinical‐grade hiPSCs into β cells in clinically compliant conditions and (3) mandatory quality control and product release criteria necessitated by various regulatory bodies to approve the use of the cell‐based products.

## Graphical abstract

Multitude of innovations had successfully revolutionized the field of regenerative medicine and the use of human induced pluripotent stem cell (hiPSC)‐derived β cells to improve the outcomes of diabetes patients is gradually becoming a reality. Here, we detail the crucial considerations for clinical manufacturing of hiPSC‐derived cells, as well as the necessary steps to obtain eventual regulatory approval of countries such as the United States, Europe, Japan and China.

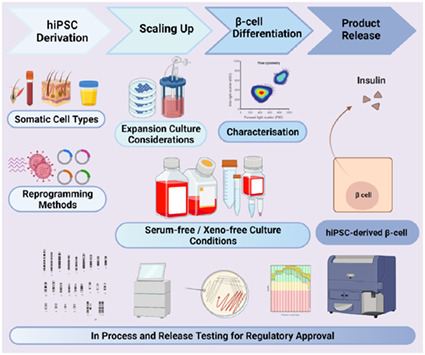

## 1 INTRODUCTION

Diabetes is a debilitating disease affecting millions worldwide.^1^ Depending on the subtype, diabetes can be attributed to the autoimmune destruction of pancreatic beta cells (β cells) in type 1 diabetes (T1D), or dysfunction of β cells in type 2 diabetes (T2D). As such, curative treatment of diabetes may be attained by β cell replacement therapy. Apart from whole pancreas or islet transplantation, β cells may also be replaced by transplanting human pluripotent stem cell (hPSC)‐derived β cells into diabetes patients to achieve insulin independence.

hPSCs are one of the most attractive sources of cells for cell therapy applications due to their unlimited proliferative capacity and their ability to differentiate into lineages of the three germ layers.^2^ While tremendous progress has been made in developing and refining strategies to differentiate hPSCs into clinically suitable insulin‐producing endocrine derivatives or functional β cells,^3^, ^4^, ^5^, ^6^, ^7^ clinical application and commercialization of these functional β cells for diabetes treatment are impeded by numerous difficulties, notably the regulatory challenges surrounding the generation, release and clinical use of stem cell‐derived products for cell therapy purposes.^8^, ^9^, ^10^, ^11^

**FIGURE 1:**
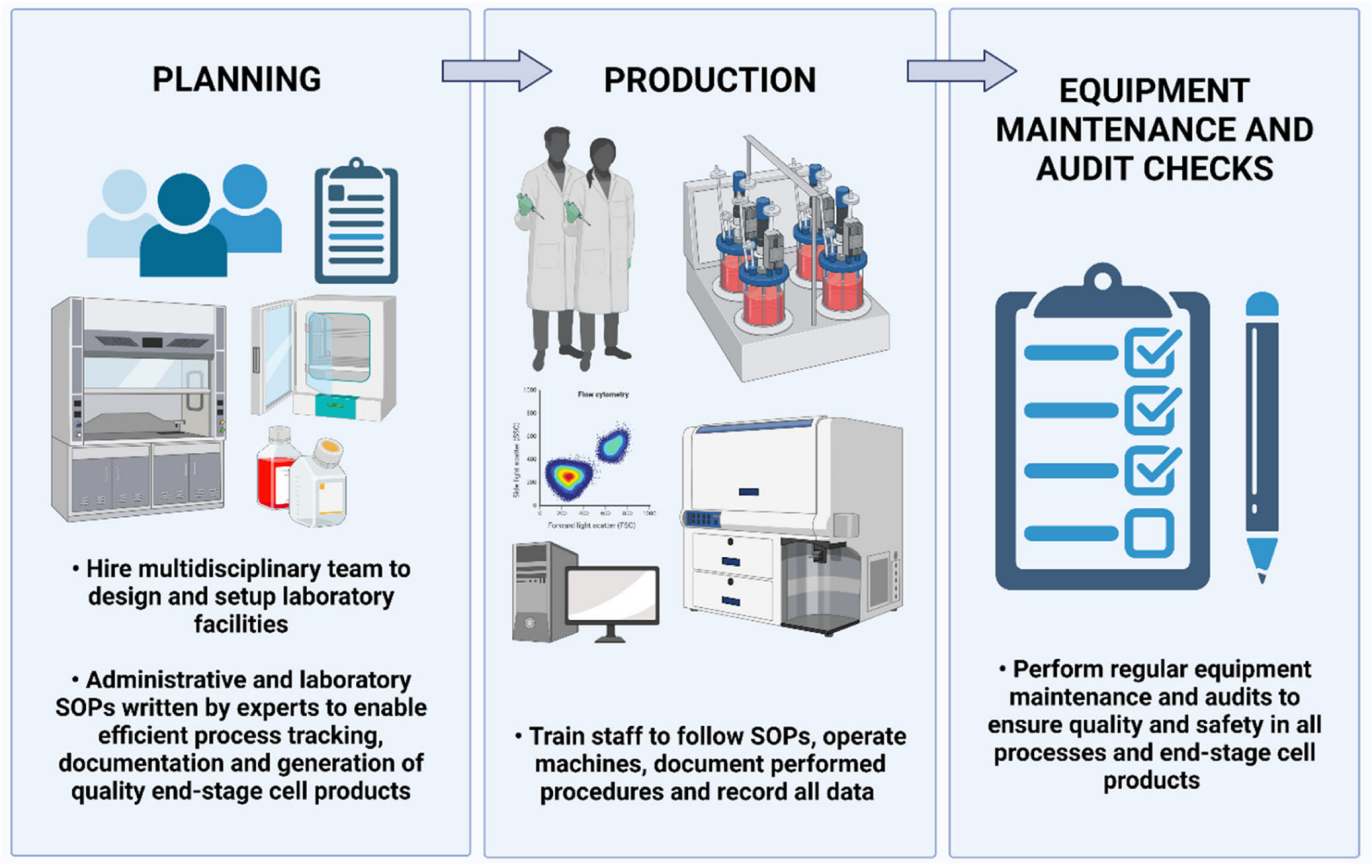
Workflow to generate clinically compliant stem cell‐based products with good manufacturing practice (GMP). First, planning of the correct facility design and processes is enabled by putting together a multidisciplinary team of stem cell biologists, process engineers and skilled laboratory managers. Standard operating procedures (SOPs) need to be devised for both administrative procedures such as procurement and shipping of raw materials, reagents and equipment and laboratory procedures such as stem cell maintenance, protocol for differentiating stem cells to end‐stage cell products, operating bioreactor systems and performing flow cytometry for cell characterization. After the planning phase and setting up of all GMP facilities and processes, staff must be trained on all relevant SOPs before proceeding with the manufacturing process. Trained staff will be required to execute the SOPs, document all their activities and observations in logbooks and record all quality control data generated. To ensure quality performance, routine equipment maintenance and on‐site audit checks by regulators on current processes, previous batch records, staff practices and hygiene will need to be conducted. Processes will need to be reviewed and improved if necessary. This figure is created with BioRender.com

In recent years, there have been various international efforts by key stem cell experts to harmonize the critical quality attributes (CQAs) of clinically compliant stem cells and stem cell‐derived cell therapy products,^12^, ^13^, ^14^, ^15^, ^16^, ^17^ but the requirements dictated by different national and international regulatory bodies and organizations (Table 1) may still vary.^18^, ^19^, ^20^, ^21^, ^22^, ^23^, ^24^, ^25^, ^26^, ^27^, ^28^ Therefore, the industry will need to assess which countries to operate in and engage the relevant regulatory authorities to ensure compliance in the entire cell product manufacturing process. Generally, the overarching principles that are important in the generation of clinically compliant stem cell‐based products that conform to good manufacturing practice (GMP) are: writing and following of site master files and standard operating procedures (SOPs), having well‐defined procurement, storage, shipment and tracking processes, incorporating proper facility design, training of staff to perform and document all laboratory and administrative processes, regular equipment maintenance and last but not least, conducting regular quality checks and compliance assessments to ensure accountability, performance and safety in all processes and end products (Figure 1).^23^, ^25^

**TABLE 1:** Non‐exhaustive list of regulatory authorities and stem cell organizations involved in stem cell therapies

Regulatory authorities		
Region	Name	Country/sub‐region
The West	Food and Drug Administration (FDA)	United States
	European Medicines Agency (EMA)	Europe
	Human Fertilization and Embryo Authority (HFEA)	United Kingdom
	Health Canada	Canada
	National Regulatory Authorities of Brazil (ANVISA)	Brazil
The East	Pharmaceuticals and Medical Devices Agency (PMDA) and Ministry of Health, Labour and Welfare (MHLW)	Japan
	National Medical Products Administration (NMPA), formerly known as China Food and Drug Administration (CFDA)	China
	Therapeutic Goods Administration (TGA)	Australia
	Ministry of Food and Drug Safety (MFDS), formerly known as the Korea Food & Drug Administration (KFDA)	Korea
	Health Sciences Authority (HSA)	Singapore
	Taiwan Food and Drug Administration (TFDA)	Taiwan
	The Department of Health‐Abu Dhabi	Abu Dhabi
Stem cell organizations		
Region	Name	Country/sub‐region
	International Society for Stem Cell Research (ISSCR)	Global
	International Society for Cell & Gene Therapy (ISCT)	Global
	International Stem Cell Banking Initiative (ISCBI)	Global
	The Global Alliance for iPSC Therapies (GAiT)	Global
European Union (EU)	EuroStemCell	Europe
	German Society for Stem Cell Research (GSZ)	Germany
	German Stem Cell Network (GSCN)	Germany
	Stem Cell Network North Rhine‐Westphalia (NRW)	Germany
	French Society for Stem Cell Research	France
	Associazione di Biologia Cellulare e del Differenziamento (ABCD)	Italy
	Danish Stem Cell Society (DASCS)	Denmark
	Norwegian Center for Stem Cell Research (NCSCR)	Norway
	Austrian Society of Stem Cell Research	Austria
	Swiss Stem Cell Network	Switzerland
	Belgian Society for Stem Cell Research (BeSSCR)	Belgium
	Irish Stem Cell Foundation	Ireland
United Kingdom (UK)	UK Stem Cell Foundation	UK
	UK Stem Cell Bank	UK
	UK Regenerative Medicine Platform	UK
Americas	California Institute for Regenerative Medicine (CIRM)	California
	New York Stem Cell Foundation (NYSCF)	New York
	Stem Cell Network (SCN)	Canada
	Canadian Stem Cell Foundation	Canada
	Associação Brasileira de Terapia Celular (Brazilian Association for Cell Therapy) (ABTCel)	Brazil
	Rede Nacional de Terapia Celular (National Network of Cell Therapy)	Brazil
Asia‐Pacific Region	Australasian Society for Stem Cell Research (ASSCR)	Australasia
	Stem Cells Australia	Australia
	The National Stem Cell Foundation of Australia (NSCFA)	Australia
	Japanese Society for Regenerative Medicine (JSRM)	Japan
	Stem Cell Society Singapore (SCSS)	Singapore
	Korean Society for Stem Cell Research (KSSCR)	Korea
	Chinese Society for Stem Cell Research (CSSCR)	China
	Taiwan Society for Stem Cell Research (TSSCR)	Taiwan
Middle‐East	Israel Stem Cell Society (ISCS)	Israel
	Regenerative and Bionic Medicine Network (RBMN) of Egypt	Egypt
	Abu Dhabi Stem Cells Center (ADSCC)	Abu Dhabi

Here, we focus on discussing human induced pluripotent stem cell (hiPSC)‐derived insulin‐producing β cells as the commercial therapeutic product for diabetes treatment. While both human embryonic stem cells (hESCs) and hiPSCs have been used for cell therapy,^2^ the derivation of hESCs is accompanied by the ethically controversial destruction of human embryos. As such, the derivation of hESCs is permitted only in parts of the United States (US), China and Europe (EU).^29^ In contrast, hiPSCs are a more universal resource for clinical and commercial purposes as they do not face the same ethical issues that hESCs face, and can also be prepared from various somatic cell types of choice.^30^

In this review, we analyse the suitability of various somatic cell reprogramming strategies to generate hiPSCs destined for clinical translation, discuss the importance of xeno‐free culture systems and summarize the mandatory CQAs of clinically compliant hiPSCs. We also provide insights into the methods and technical complexities involved in the scale‐up of clinical‐grade hiPSCs before differentiating them into insulin‐producing β cells—a process that is technically challenging but vital for the success of large‐scale commercial cell manufacturing.^31^ Furthermore, we offer suggestions on key parameters for quality control (QC) testing during specific stages in the differentiation process and provide a summary of the current efforts on transitioning to serum‐free and/or xeno‐free differentiation culture medium to rule out the possibility of animal‐derived infections in future transplant patients. Last but not least, we present a broad overview of the guidelines governing the release of hiPSC‐derived β cells for clinical applications in diabetes treatment, detailing the extensive testing required by international regulatory bodies to ascertain product identity, viability, sterility, safety and potency before product release.

## 2 GENERATION, CULTURE, QC TESTING AND CHARACTERIZATION OF CLINICAL‐GRADE HIPSCS

### 2.1 Reprogramming strategies

The first major step in generating hiPSCs of clinical grade is to select a suitable and safe reprogramming approach for cell therapy applications. Successful reprogramming of human skin fibroblasts into hiPSCs with the induction of four transcription factors (*OCT3/4*, *SOX2*, *c‐MYC* and *KLF4*) using retroviral transduction was first achieved in 2007 by Takahashi and Yamanaka.^32^ However, the use of retroviruses causes undesired permanent integration of viral vector transgene and backbone into the genome, thus raising concerns pertaining to the risk of unintended insertional mutagenesis.^32^

Since then, safer reprogramming alternatives to generate clinical‐grade hiPSCs have been developed. Amongst these, the use of the Sendai virus (SeV), episomal vectors and synthetic mRNA are some of the most efficient reprogramming methods and will be discussed in detail below. A comparison of reprogramming approaches with SeV, episomal vectors and synthetic mRNA is also summarized in Table 2.

**TABLE 2:** Comparison of Sendai virus, episomal and mRNA reprogramming methods for clinical and commercial use

	SeV	Episomal	mRNA
Suitable starting cell types and reported efficiency	High efficiency—blood,^33^ urine,^34^, ^35^ hair keratinocyte^36^ Moderate efficiency—skin fibroblast^37^	High efficiency—blood,^33^ urine,^38^ hair keratinocyte^39^, ^40^ Low efficiency—skin fibroblast^37^	High efficiency—skin fibroblast,^37^ urine^41^ Low efficiency—blood, not efficient but possible with blood‐derived endothelial progenitor cells,^42^ hair keratinocytes^43^
Examples of xeno‐free methods described	Churko et al.^44^ and Macarthur et al.^45^	Chen et al.^46^	Warren et al.^47^, ^48^
Reprogramming agent clearance	Within ~10 passages^45^	Within ~11–20 passages^49^	Immediately
Ease of assimilation into clinical processes	No risk of genome integration but uses virus^50^	Does not use virus but holds some risk of episomal vector genome integration^51^	No known issues
Source companies with rights to reprogramming kits (non‐exhaustive list)	Thermo Fisher Scientific (CytoTune‐iPS Sendai Reprogramming Kit)	Thermo Fisher Scientific (Epi5 Episomal iPSC Reprogramming Kit) Lonza (Lonza L7 hiPSC Reprogramming and hPSC Culture System) Alstem (Episomal iPSC Reprogramming Kit) Creative Bioarray (QualiStem Episomal iPSC Reprogramming Kit)	Reprocell (Stemgent StemRNA 3rd Gen Reprogramming Kit) Creative Bioarray (QualiStem RNA iPSC Reprogramming Kit) Stem Cell Technologies (ReproRNA‐OKSGM) Merck (Simplicon RNA Reprogramming Kit)
Labour requirement	One‐time administration of SeV^50^	One‐time transfection of episomal vectors^51^	Repeated administration of mRNA daily till colony emergence^52^

As SeV is a single‐stranded negative‐sense non‐integrative RNA virus that does not replicate through the DNA phase, SeV reprogramming strategies thus harbour no risk of genome integration. Its use in reprogramming was first reported in 2009 by Fusaki et al.,^50^ and many studies have demonstrated high efficiency in reprogramming multiple somatic cell types into hiPSCs.^37^, ^44^, ^50^ However, it is to be noted that the use of virus in SeV‐based reprogramming complicates the regulatory authority approval process, and there is a need to dilute and eventually rid hiPSCs of residual SeV via passaging.^37^, ^51^ Based on Macarthur et al.,^45^ complete vector clearance can be achieved without complications within ~10 passages after SeV reprogramming.

In contrast, the use of plasmid DNA in episomal reprogramming renders ease of assimilating this method into clinically compliant processes as compared to SeV reprogramming.^37^, ^51^ While episomal reprogramming efficiency is poorer than that of SeV, several groups have developed techniques that improved the efficiency of this method of reprogramming.^46^, ^49^, ^53^, ^54^ It is notable that one drawback of the episomal method is that there remains a low possibility of episomal vector integration into the genome.^37^, ^51^

Out of the three methods, the use of synthetic mRNA is possibly the safest for clinical translation as it does not utilize virus or plasmid DNA that carries an inherent risk of genome integration.^52^ However, its main limitation is its poorer efficiency in reprogramming some non‐invasive cell sources such as blood and keratinocytes (Table 2). Furthermore, this method is laborious as mRNAs must be fed to the cells daily until colony emergence.^37^, ^52^ Therefore, this method is mainly recommended when skin fibroblasts are used as the starting somatic cell type.

### 2.2 Somatic cell types suitable for reprogramming

Blood cells are generally prioritized over skin fibroblasts as the starting somatic cell type for reprogramming due to the ease of accessibility.^55^, ^56^, ^57^ Furthermore, skin fibroblasts harbour somatic mutation risks due to outward exposure to environmental mutagens such as sunlight.^58^ Other non‐invasive cell sources such as exfoliated renal epithelial cells from urine and keratinocytes from hair can also be considered if desired.^59^, ^60^ Ultimately, the choice of reprogramming method and starting somatic cell type will be based on the industry's preference when aligned with the regulatory requirements of their site of operation.

### 2.3 Xeno‐free culture conditions

The use of xeno‐free culture reduces the risk of immune reactions and zoonotic infections associated with the use of animal‐derived reagents. The manufacturing process should thus be designed in which the entire pipeline is done in chemically defined xeno‐free culture systems. In addition, all cell culture components should be defined chemically for standardization when culturing hiPSCs, for documentation purposes and for preventing issues arising from lot‐to‐lot variability in undefined components such as fetal bovine serum (FBS).^61^, ^62^, ^63^, ^64^, ^65^

Currently, xeno‐free conditions have been successfully incorporated into SeV, episomal and synthetic mRNA reprogramming processes by several groups.^44^, ^45^, ^46^, ^47^, ^48^ A multitude of xeno‐free stem cell culture media and chemically defined feeder‐free extracellular matrix proteins, such as vitronectin and laminin, are now commercially available to replace feeder cells and Matrigel in the surface coating of hiPSC culture dishes. These should all be integrated into the production process.^44^, ^45^, ^46^, ^47^, ^48^, ^61^, ^62^, ^64^, ^65^, ^66^, ^67^, ^68^, ^69^, ^70^, ^71^, ^72^, ^73^, ^74^ Finally, the hiPSC passaging reagent should also be carefully chosen based on efficiency, reliability and reproducibility.^62^, ^71^, ^74^

### 2.4 Characterization of clinical‐grade hiPSCs

Following the reprogramming of somatic cells to hiPSCs, it is crucial for hiPSCs to undergo mandatory QC testing and characterization to ascertain their CQAs as high quality clinical grade hiPSC lines. These tests are designed to check for (1) sterility, (2) purity, (3) cell viability, (4) genomic identity/stability and (5) pluripotency (Table 3). For sterility and purity testing, hiPSCs must test negative for bacteria, virus and mycoplasma, and should be free of endotoxins. Post‐thawing, the viability per vial of hiPSCs should be minimally 50% but stricter criteria of 60%–80% have also been imposed.^61^, ^75^, ^76^

**TABLE 3:** Summary of relevant in‐process and final product testing during hiPSC‐derived β‐cell manufacturing

Product testing	Stage to conduct	Assays	Criteria
Sterility	Working cell bank	Routine testing with compendial 14‐day sterility test and 28‐day mycoplasma test	Free of adventitious agents
	Before starting β‐cell differentiation		
	Final product	PCR‐based mycoplasma testing ATP bioluminescence measurement of filtered samples CO_2_ monitoring system of filtered samples	
Purity	Before starting β‐cell differentiation	Endotoxin test	Free from endotoxin (<0.25 EU/ml)
	Final product	Endotoxin test ELISA assay for growth factors and cytokines	Free from endotoxin (<0.25 EU/ml), cytokines and other growth factors
Viability and cell count	Post‐thawing of hiPSCs	Trypan blue staining for viability Manual cell counting with the use of a haemocytometer	Cell viability and number is dependent on cell line and number of cells frozen per vial
	Before starting β‐cell differentiation		
	Final product		Aggregate size of 100–250 μm Minimally 70% of viable cells per aggregate Free of adventitious agents
Identity	Working cell bank	STR fingerprinting analysis Flow cytometry of pluripotency markers (e.g., TRA‐1‐60, OCT4, SOX2, NANOG) Teratoma assay or other appropriate pluripotency test	No cross‐contamination of other cell lines Minimally >70% pluripotency marker expression hiPSCs are able to generate teratoma when transplanted in vivo or demonstrate formation of three germ layers
	During β‐cell differentiation	*Undifferentiated hiPSCs*: >80% OCT4^+^ and TRA‐1‐60^+^ *(optional*: *SOX2, NANOG, SSEA4)* *Definitive endoderm*: >80% CXCR4^+^ and SOX17^+^ *(optional*: *FOXA2)* *Primitive gut tube*: *optional testing for FOXA2*, *HNF1B*, *HNF4A* *Pancreatic progenitor*: >60% PDX1^+^ and NKX6.1^+^ *(optional: HNF6*, *SOX9*, *PDX1)* *Endocrine progenitor*: *optional testing for NGN3*, *NEUROD1*, *PAX4*, *NKX6.1*, *CHGA*	
	Final product	>40% INS^+^ and NKX6.1^+^ or >20% C‐peptide^+^ and NKX6.1^+^ *(optional*: *CHGA*, *MAFA)*	
Potency	Final product	GSIS Calcium flux assay	Functional GSIS activity comparable to human islets Calcium influx in response to high glucose levels
Safety	Before starting β‐cell differentiation	Karyotype analysis by G‐banding Whole genome/exome sequencing	Normal karyotype No chromosomal abnormalities
	Final product	Karyotype analysis by G‐banding TRAP assay for telomerase activity Flow cytometry and qRT‐PCR of pluripotency markers (e.g., TRA‐1‐60, OCT4, SOX2, NANOG)	Normal karyotype No telomerase activity No proliferative activity No residual pluripotency cell

To ascertain genomic identity/stability, the clearance of residual reprogramming vector and/or virus in seed and master cell banks must be proven by appropriate methods.^77^, ^78^ Karyotyping analysis by Giemsa banding (G‐banding) should also be performed to confirm with 95% probability that the hiPSCs do not carry chromosomal abnormalities and where possible, whole genome or exome sequencing can also be performed.^61^, ^78^ To distinguish between hiPSC lines and to avoid cross‐contamination, short tandem repeat (STR) fingerprinting analysis should be performed with minimally eight core STR loci according to International Cell Line Authentication Committee guidelines,^19^ while minimally 15 loci are required if hiPSCs are destined for autologous cell therapy.^79^ In Lonza's cell line authentication process, as many as 16 loci are usually analysed for at least 80% match.^80^

To assess pluripotency, flow cytometry analysis is a robust quantitative method to determine pluripotency marker expression in hiPSC lines. A combination of surface pluripotency markers (e.g., TRA‐1‐60) and intracellular pluripotency markers (e.g., OCT3/4, SOX2, NANOG) should be selected for flow cytometry, and >70% pluripotency marker expression can be set as the minimum standard for QC testing.^75^ Last but not least, pluripotency tests such as the teratoma assay or embryoid body‐based three germ layer differentiation will also be ideal to demonstrate pluripotency of the hiPSCs.^77^, ^78^

## 3 TECHNICAL CONSIDERATIONS FOR THE EXPANSION AND DIFFERENTIATION OF HIPSCS INTO INSULIN‐PRODUCING Β CELLS

Following the generation of clinical‐grade hiPSCs, these hiPSCs must then be expanded to generate sufficient biomass before differentiation into insulin‐producing β‐cells, as it is difficult to scale up during the differentiation process. About 5000–10,000 islet equivalent (IEQ) per kilogram of the recipient's body weight is required to improve metabolic control of blood glucose levels.^81^, ^82^ As ~1000 β cells are estimated to be present in an IEQ,^83^ close to 1 billion hiPSC‐derived β cells will be required for each diabetes transplant patient. Similarly, a therapeutic dose of more than 1 billion cells per patient is also commonly estimated by pharmaceutical manufacturers in the context of large‐scale commercial manufacturing for allogeneic cell therapy.^31^

**FIGURE 2:**
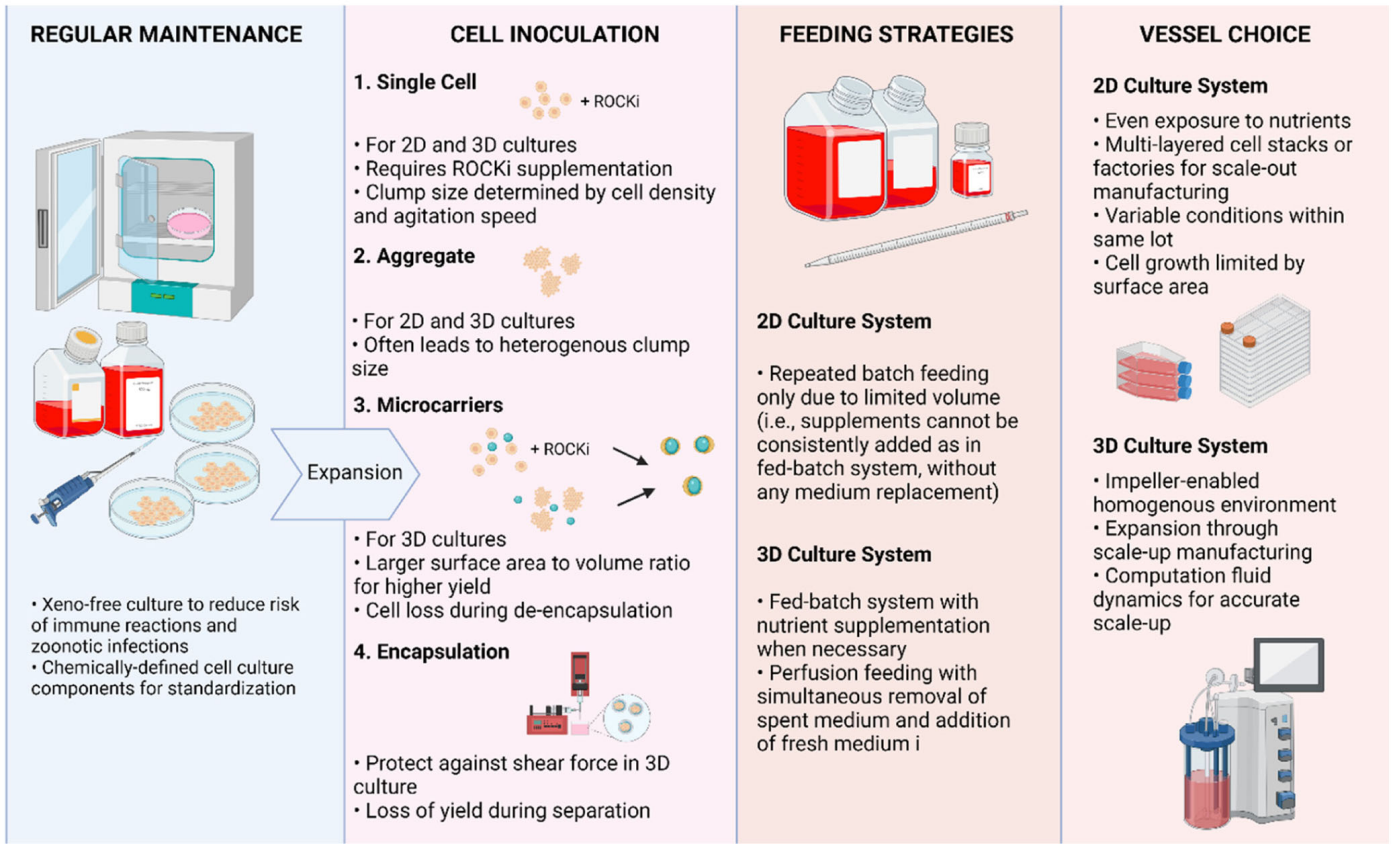
Technical considerations for the expansion and differentiation of human induced pluripotent stem cells. Technical considerations include the cell inoculation method, feeding strategies and choice of two‐dimensional (2D) or three‐dimensional (3D) culture vessel. In general, 3D culture systems have a wider range of cell inoculation methods and feeding strategies that are applicable, and are more scalable than the 2D static counterpart. This figure is created with BioRender.com

Hence, to cater to the demand for hiPSC‐derived β cells, hiPSCs, which are the starting material for the differentiation process, will need to be readily expanded. This therefore underlines the need for optimized culture conditions and standardized vessels for robust manufacturing of cell‐based products and guarantee the production of sufficient hiPSC‐derived β cells for cell therapy. To that end, various strategies pertaining to the inoculation methods and feeding strategies for two‐dimensional (2D) static culture system and three‐dimensional (3D) suspension‐based conditions, as well as the vessel choices for hiPSC expansion (Figure 2) are described in more detail below.

### 3.1 Cell inoculation of hiPSCs

To increase the viability and yield of hiPSCs in expansion culture, cell inoculation has been carried out in a myriad of ways—single cell inoculation, aggregate inoculation, inoculation with microcarrier‐bound hiPSCs and inoculation with microencapsulated hiPSCs. As hiPSCs cannot survive as single cells, single cell inoculation of hiPSCs will require an initial supplementation with Rho‐associated, coiled‐coil containing protein kinase inhibitor (ROCKi).^84^ By modulating the cell density inoculated for both 2D and 3D culture systems, and the agitation speed of suspension cultures, cell aggregate size can be further refined to optimize cell viability and hiPSC expansion.

In the past, aggregate inoculation methods relied on mechanical splitting or enzymatic dissociation of stem cell colonies.^85^, ^86^ As sizes of starting aggregates often differ, subsequent clumps were often heterogeneous and could affect the efficiency of downstream β‐cell differentiation if this method were used for hiPSC expansion.^87^ To circumvent this, single cell dissociation‐based aggregate inoculation method can be utilized, where hiPSCs are first dissociated into single cells and incubated to form homogeneous aggregates in suspension, before being transferred to the expansion culture. Using this approach, Borys et al.^88^ successfully generated uniform hiPSC spheroids (214 ± 6 μm) with high proliferative capabilities (32‐fold increase over 6 days). The homogeneity of spheroid sizes generated will also improve differentiation efficiency, consistency, functionality and aid in downstream clinical applications of hiPSCs.^89^

To further increase the yield of 3D suspension cultures, microcarriers have also been added in tandem with hiPSC inoculation. As the use of uncoated microcarrier beads result in gradual loss of pluripotency and diminished cell growth when hiPSCs are continuously passaged,^90^ microbeads surface‐coated with xeno‐free and defined matrices that are GMP compliant have been developed and can be adopted for hiPSC expansion.^91^, ^92^, ^93^ Due to the increased surface area to volume ratio, microcarrier‐based suspension cultures have consistently led to higher expansion yield than 2D cultures.^94^, ^95^ However, the expansion of hiPSCs on microcarriers can be problematic for clinical applications, as microcarriers should be separated from the final cell harvest to comply with regulatory demands, inevitably leading to cell loss. Nonetheless, improvements in separation methods have been made and the development of dissolvable matrices are currently being explored,^96^, ^97^ positioning microcarrier 3D suspension systems as a plausible alternative for large‐scale manufacturing in the future.

Last but not least, to protect cells against the inherent shear force of suspension cultures, hiPSCs can be microencapsulated in hydrogel prior to inoculation,^98^, ^99^ with biocompatible hydrogels such as alginate and agarose used.^100^, ^101^, ^102^ However, such methods prove cumbersome in bioprocessing as additional re‐encapsulation and de‐encapsulation steps will be required for routine hiPSC passaging.^103^ In addition, nutrient diffusion and monitoring of cell growth in the capsule may also be limited by the physical properties of encapsulation.

### 3.2 Feeding strategies

Unlike 2D planar systems where limited culture size will require frequent medium replacement (repeated batch feeding), 3D vessels with larger volume capacity and capability for automated processing can allow for fed‐batch systems and perfusion systems to be implemented. In fed‐batch systems, nutrient supplements can be added to prevent growth inhibition of hiPSCs. In contrast, spent medium is continuously removed while fresh medium is simultaneously added in perfusion systems. Overall, perfusion feeding is more advantageous as it allows for more homogeneous culture conditions and perfusion feeding has in fact been previously documented to lead to 47% higher expansion yield than batch‐fed cultures.^104^ In addition, as a closed‐loop system, perfusion feeding strategies also minimize the risk of contamination and thus, reduce the number of in‐process sterility tests.^105^ However, the overall operational complexity and costs associated with perfusion systems make it prohibitive for widespread adoption.^106^

### 3.3 2D versus 3D culture vessels for cell culture and differentiation

Despite the incompatibilities of 2D planar culture with some of the afore‐mentioned inoculation strategies, there are some merits to this method. 2D static culture system has been routinely employed in laboratories for stem cell maintenance as it is simple to implement and is more cost‐effective than its 3D suspension‐based counterpart. Culturing hiPSCs as a monolayer, rather than aggregates in suspension, also allows the cells to be more evenly exposed to nutrients. In the 2D culture system, multi‐layered cell stacks and factories can be utilized for the expansion of hiPSCs through a scale out process, where capacity increases linearly with the number of cell stacks added.^107^, ^108^

However, as cell growth will still be limited by the surface area of culture plates, 3D culture system is a more viable alternative for hiPSC expansion. Moreover, variability in conditions between cell stacks of the multi‐layered cell plates have also been documented.^108^ Thus, homogeneity of hiPSC quality within the same lot may be difficult to maintain when multi‐layer cell stacks or factories are used for clinical manufacturing. Therefore, 3D suspension cultures, where large volumes of cells can be cultured in an impeller‐enabled homogenous environment, are typically preferred for scale‐up manufacturing. Of the 3D culture systems, stirred tank bioreactors are most commonly employed in pharmaceutical production due to their capability to accommodate large volumes,^109^ although other 3D suspension culture vessels with different rotational methods have also been devised.^85^, ^110^ Equipped with a horizontal blade at the bottom, the rotational rate of the impeller in stirred tank bioreactors can be modified based on bioreactor volume to facilitate nutrient diffusion and oxygen mass transfer.^111^ However, current methods of scale‐up for stirred tank bioreactors are based on empirical equations and are subjective depending on the geometry of the vessel system and impeller design, resulting in inaccurate recapitulation of the local flow pattern within the bioreactor.^109^ Hence, computational fluid dynamics should be utilized for a more accurate scale‐up in bioprocessing, such that further adjustment of cell culture parameters in multiple iterations can be avoided.^112^, ^113^

## 4 DIFFERENTIATION METHODS, CULTURE CONDITIONS, STRATEGIES TO EXCLUDE UNDIFFERENTIATED HIPSCS, QC AND CHARACTERIZATION DURING Β CELL DIFFERENTIATION

### 4.1 Methods to differentiate hiPSCs into β cells

Following successful scale‐up of hiPSCs, the cells will be progressively differentiated along the pancreatic lineage to form insulin‐producing β cells. The differentiation process can be broadly broken down into five developmental stages (definitive endoderm, primitive gut tube, pancreatic progenitor, endocrine progenitor and insulin‐producing β cells), and methods to differentiate hPSCs into pancreatic β cells often involve the timed addition of various growth factors and small molecules to model pancreatic development.^3^, ^4^, ^5^, ^114^, ^115^, ^116^, ^117^, ^118^, ^119^, ^120^

The first widely‐employed step in differentiating hPSCs to pancreatic cells is definitive endoderm commitment. In 2005, D'Amour et al.^121^ devised an efficient method directing up to 80% of hPSCs to the definitive endoderm lineage via the addition of activin A and low serum. Protocols demonstrating further specification and differentiation of hPSCs to PDX1^+^NKX6.1^+^ pancreatic progenitors were subsequently published.^4^, ^122^, ^123^ In 2014, Pagliuca et al.^114^ and Rezania et al.^3^ reported successful in vitro generation of NKX6.1^+^C‐peptide^+^/NKX6.1^+^INS^+^ functional hPSC‐derived β cells at ~40% and ~50% efficiency, respectively. As the differentiation is not 100% efficient, it is vital for manufacturers to account for the losses through the scale‐up of hiPSCs. Despite these cells having reduced glucose‐stimulated insulin secretion (GSIS) functionality compared to human islets, importantly, the cells led to the reversal of diabetes in diabetic mice post‐transplant. Specifically, Rezania et al.^3^ added vitamin C to the cells in the early differentiation stages to generate pancreatic progenitors co‐expressing PDX1 and NKX6.1 before adding a combination of growth factors and small molecules such as ALK5 inhibitor (ALK5i), BMP receptor inhibitor and thyroid hormone (T3) to induce co‐expression of PDX1, NKX6.1, NEUROD1 and NKX2.2 in the cell population. Finally, Notch inhibitor was added to direct the PDX1^+^NKX6.1^+^NEUROD1^+^ cells to express insulin.^3^ Pagliuca et al.^114^ also used a similar approach where different small molecules and growth factors were added to hPSCs at specific time points, though the chemicals used varied from Rezania et al.'s method.

More recently, modified methods were able to generate cells with more robust and dynamic GSIS, overcoming the limitation of poor GSIS in past protocols.^6^, ^87^, ^124^ Nair et al.^5^ first showed that isolating INS^+^ cells at an early immature stage (Day 20 of differentiation) via fluorescence‐activated cell sorting (FACS), followed by reaggregation of the sorted INS^+^ cells into clusters in vitro leads to enhanced β‐cell maturation. However, while dynamic GSIS was observed, the second‐phase response was not sustained. Meanwhile, Velazco‐Cruz et al.^87^ reported that selective modulation of transforming growth factor‐β signalling coupled with the resizing of cell clusters at the final stage of differentiation helps to obtain cells capable of dynamic GSIS with a more sustained second‐phase response. While the functionality of hPSC‐derived β cells still pale in comparison to human islets, these continued improvements in protocols are crucial for clinical development of hiPSC‐derived β cells.

Despite the afore‐mentioned progress, one key limitation of current differentiation methods is that the entire process, or at least part of it, is not serum‐free and/or xeno‐free. This may lead to translational issues which we will address in the next subsection.

### 4.2 Current serum‐free and/or xeno‐free standards incorporated into β‐cell differentiation methods

As an important source of various complex growth factors and molecules, FBS is often included as an essential nutrient supplement in culture media supporting the differentiation into pancreatic cells.^4^, ^5^, ^115^, ^116^, ^120^, ^125^, ^126^ While most regulatory bodies do not explicitly ban the use of FBS in the manufacturing process of stem cell‐derived cell products, the undefined nature of FBS with batch‐to‐batch variation may increase the complexity of QC and safety testing required.^127^ Combined with the risks of disease transmission and ethical issues associated with its zoonotic origin, there is a need to move away from FBS towards other serum‐free and xeno‐free defined alternatives for hiPSC differentiation.^127^

A popular serum‐free defined alternative adopted by various groups to obtain hiPSC‐derived β cells is bovine serum albumin (BSA), as it is an abundant protein present in serum, even though it is not strictly considered xeno‐free, being a bovine‐derived protein.^3^, ^6^, ^7^, ^114^, ^128^ The use of supplements such as B‐27, which are commercially available in both serum‐free and/or xeno‐free versions, have also been reportedly added during β‐cell differentiation, though these protocols did not completely eliminate FBS use.^5^, ^115^, ^128^ This partial serum‐free replacement approach has also been utilized in Pagliuca et al.,^114^ where differentiation media in the first 20 days of differentiation (S1, S2, S3, S5 media) is supplemented with fatty‐acid free BSA instead of FBS. However, the basal CMRL‐1066 medium for S6 media used in the final 15 days of differentiation, involving committing endocrine progenitors to the functional insulin‐producing β‐cell fate, is still supplemented with 10% FBS.^114^ Separately, there have also been some efforts in recent years to replace S6 media, such as the use of an enriched serum‐free media designed to ensure a serum‐free differentiation process.^87^

Separately, Rezania et al. and several other groups were reportedly able to completely replace FBS with BSA supplementation in their β‐cell differentiation protocol.^3^, ^7^, ^126^, ^128^ While the complete elimination of FBS supplementation is a huge step towards serum‐free and xeno‐free differentiation conditions, it is notable that the basal MCDB131 medium commonly adopted for β‐cell differentiation still contains low amounts of dialyzed FBS.^129^ Until a completely serum‐free and/or xeno‐free GMP compliant β‐cell differentiation process is devised, it is currently recommended for manufacturers to adopt minimal serum use in their β‐cell differentiation procedure to lower the risks of spreading zoonotic disease and infections post‐administration. Thus, should low amounts of serum components such as FBS need to be used for β‐cell differentiation, safety and sterility testing in end‐stage cell products should be rigorously implemented before clinical applications.

Eventually, to completely eliminate animal‐derived components in differentiation media, human serum albumin may be used as a replacement for BSA.^130^ Furthermore, apart from transitioning to differentiation media with minimal serum supplements, other plausible strategies such as using human recombinant growth factors or small molecules devoid of any animal‐derived component can be used. However, the change has to be thoughtfully considered, selected, tested by manufacturers and vetted and approved by regulatory bodies before incorporating into the finalized hiPSC‐derived β‐cell differentiation procedure.^131^

Finally, in relation to purity, it will also be necessary to demonstrate that cytokines and growth factors used during the differentiation process are not present in the final product.^132^ Endotoxin testing will also be required. While an acceptable range of endotoxin levels has not been set for cell therapy products, a maximum endotoxin level of 0.25 EU/ml has been established for water in injection products and may be extrapolated for transplantable hiPSC‐derived β cells.^133^

### 4.3 Methods to exclude residual hiPSCs from β‐cell population during differentiation

Despite the tremendous potential of hiPSC‐derived β cells for diabetes treatment, the tumourigenicity of residual undifferentiated hiPSCs following the differentiation process poses a critical safety risk in cell therapy applications.^134^ It is therefore important to implement strategies that can eliminate undesirable remaining hiPSCs in the differentiated population destined for cell therapy use. There are a variety of techniques (genetic, chemical, antibody‐based, immunological) devised over the years to exclude hiPSCs in the final cell therapy product. Genetic methods include the use of lentiviral transduction of suicide genes such as caspase‐9 (iC9) into hiPSCs, which enables the eradication of transduced hiPSCs or any formed tumours after addition of a specific chemical inducer of dimerization that activates iC9.^135^, ^136^ One example of chemical methods is the use of survivin inhibitor YM155, which can induce apoptotic cell death of hPSCs without damaging the functionality of differentiated cells.^137^ For antibody‐based methods, Choo et al.^138^ reported the use of cytotoxic antibody that strongly selects and induces cell death in specifically undifferentiated hESCs. Finally, one example of immunological methods includes separating pluripotency marker‐expressing undifferentiated hESCs such as SSEA4 and TRA‐1‐60 from the differentiated population using magnetic‐activated cell sorting (MACS) and FACS.^139^

However, it is notable that each technique comes with its own safety concerns and limitations. For genetic modification involving the introduction of suicide genes into hiPSCs via lentiviral transduction, techniques involving viral vectors may increase tumourigenicity risk,^140^ though the problem may be alleviated in the future if novel genetic engineering strategies that do not involve viral vector use were to be developed. On the other hand, the addition of chemicals to kill undifferentiated hiPSCs raises the question of how specific the chemical is, whether the chemical is also toxic to other cell types and whether there is a need to continually expose the patient receiving cell therapy to the drug post‐transplant.^141^ Finally, sorting methods like MACS and FACS may not be 100% efficient and any remaining hiPSCs that are not sorted out and transplanted unintentionally can still lead to tumour formation, undermining the patient's health. For instance, Fong et al. reported MACS sorting of SSEA4^+^TRA‐1‐60^+^ undifferentiated hESCs at ~80% efficiency, which is a high efficiency rate yet clearly imperfect at sorting out all unwanted hPSCs.

Ultimately, it is recommended for manufacturers to weigh the pros and cons of each method and choose the most suitable method aligned to industry standards. Even with the steps to remove undifferentiated hPSCs, after each batch of hiPSC‐derived β cell is produced, testing for any tumourigenicity due to residual hiPSCs is still required. Details on product release testing and criteria based on international regulations will be discussed in the next section under the safety subsection.

### 4.4 QC and characterization based on identity during β‐cell differentiation

**FIGURE 3:**
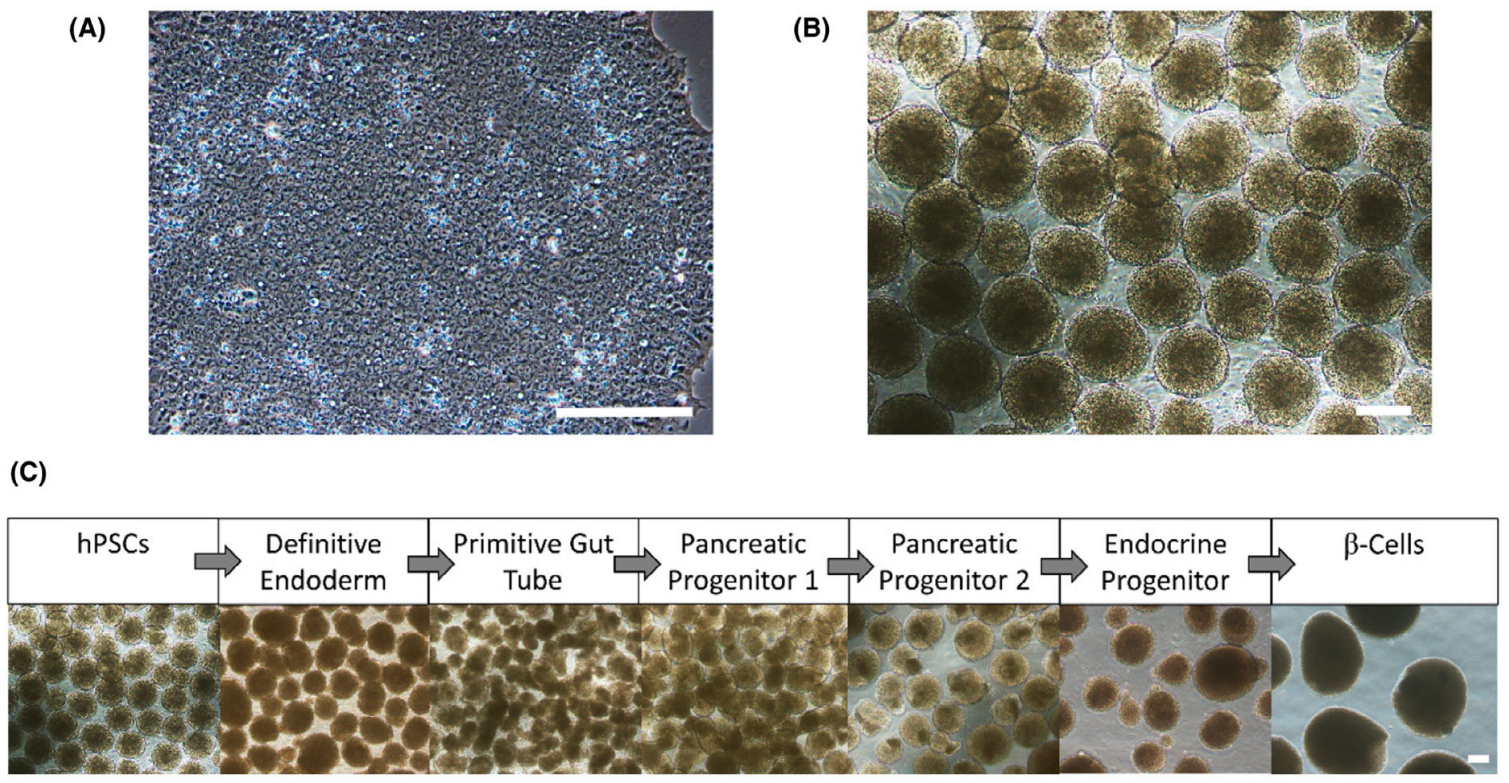
Differentiating human pluripotent stem cells (hPSCs) into insulin‐producing pancreatic β cells. (A) The gross morphology of hPSCs. (B) hPSCs in three‐dimensional cell clusters (~200 μm) dissociated from two‐dimensional monolayer culture to prepare for large‐scale differentiation in bioreactors. (C) Images of cell clusters at various stages of β‐cell differentiation, ultimately forming insulin‐producing β cells. Scale bar = 200 μm

To reduce heterogeneity, increase cell survival and maximize differentiation outcomes when clinical‐grade hiPSCs are differentiated in 3D suspension cultures as cell clusters (the current preferred choice for generating insulin‐producing β cells as mentioned in the previous section), hiPSCs with a high nuclear‐to‐cytoplasmic ratio should be used (Figure 3A). One QC strategy is to monitor and standardize the starting hiPSC clump sizes.^88^, ^114^, ^125^ Single cell‐based dissociation method as above‐mentioned can be utilized to increase homogeneity. An example of uniform hiPSC clump sizes at ~200 μm in diameter is shown in Figure 3B and the morphology of clumps during the differentiation process is shown in Figure 3C.

Another QC strategy is to characterize the cells during the hiPSC differentiation process. It is paramount to check the quality, the identity of intermediate cell types as well as the final β‐cell product manufactured. Hence, flow cytometry analysis of cell populations at various stages of differentiation with stage‐specific markers should be conducted to characterize the cell product.^132^ The flow cytometry analysis of hiPSCs and cells at selected stages during pancreatic differentiation should be performed with at least two stage‐specific markers. As gating strategies are operator‐dependent, gene expression profiling of the product in accordance to disallowed gene and gene expression of mature β cells,^142^, ^143^ along with additional data from RT‐qPCR and immunostaining of other relevant markers can also be used to supplement final product characterization by flow cytometry.

Suggestions of both mandatory and optional QC parameters during the pancreatic differentiation process are summarized in Table 3.^3^, ^6^, ^114^, ^115^, ^116^, ^128^, ^144^, ^145^ It is notable that while some protocols reportedly generate up to 90% PDX1^+^NKX6.1^+^ double positive pancreatic progenitor cells,^144^, ^146^ the bar is currently calibrated at 60% at the afore‐mentioned stage in Table 3 based on multiple considerations such as the variability in differentiation efficiencies between different hPSC lines.^3^, ^144^ As β‐cell differentiation methods are optimized to become more efficient and less cell‐line dependent over time, it is prudent for manufacturers to anticipate increasingly stringent QC parameters for stage‐specific markers as the field advances.

## 5 PRODUCT RELEASE TESTING AND CRITERIA BASED ON INTERNATIONAL REGULATIONS

Testing of the final cell therapy product is required to demonstrate its identity, purity, sterility, viability, safety and potency. While QC methods and release testing to ensure proper identity and purity of hiPSC‐derived β cells have been addressed earlier, the appropriate assays to document sterility, viability, cell count, safety and potency of hiPSC‐derived β cells are not the same. Here, we detail the various tests necessary to meet the remaining criteria for product release.

### 5.1 Sterility

As generating β cells from hiPSCs requires multiple manipulations and as they are cultivated for a relatively long duration in vitro, sterility testing of the culture environment and in‐process products should be incorporated into the acceptance criteria for release. Since the final hiPSC‐derived β cell product cannot be terminally sterilized, sterility testing of the final product is ever more essential to ensure that it is free of adventitious agents prior to administration into recipients. With reference to regulatory policies from countries such as US, EU, Japan and China,^147^, ^148^, ^149^, ^150^, ^151^ mandatory tests for virus, bacteria and mycoplasma contamination should be regularly administered during the process of derivation of hiPSC‐derived β cells and on the final product. A summary of the sterility tests to be implemented at various derivation steps for hiPSC‐derived β cells is provided in Table 3, and the methods for bacterial and mycoplasma testing will also be further discussed below.

In accordance to The International Pharmacopoeia that was harmonized with the US Pharmacopoeia, European Pharmacopoeia and Japanese Pharmacopoeia, a 14‐day sterility test and a 28‐day mycoplasma test should be administered to certify that the product is free from bacterial and mycoplasma contamination, respectively.^152^ Unfortunately, long‐term in vitro cultivation of stem cell‐derived β cells has been shown to result in decreased functionality.^87^ Hence, due to the probable short shelf‐life of hiPSC‐derived β cells, these conventional testing methods with long read‐out durations are less feasible for final product release testing.

It is noted that the US Food and Drug Administration (FDA) and European Medicines Agency (EMA) also permit product administration before final product sterility test results are obtained if justifiable QC can be conducted.^147^, ^148^ While the EMA did not specify the sterility assurance required, the FDA outlined it as (1) sterility test results on cultures sampled 48 or 72 h prior to final harvest as proxy, (2) Gram staining or other rapid detection methods conducted on representative samples of the final product lot and (3) an investigational plan for sterility failure and medical management of recipient of contaminated product.^148^

Despite relative flexibility from the FDA and EMA on sterility testing for lot release, other regulatory bodies such as the Japan Pharmaceuticals and Medical Agency (PMDA) do not permit the administration of the final product before it is certified to be free of contaminating agents.^153^ Hence, methods with shorter testing duration such as rapid microbial test should be conducted in lieu of traditional pharmacopoeia tests. The use of these methods (Table 3) is generally supported by FDA, EMA, PMDA and China National Medical Products Administration (NMPA) provided that better or equivalent sensitivity to conventional pharmacopoeia methods is demonstrated.^23^, ^154^, ^155^, ^156^, ^157^

### 5.2 Viability and cell count

To ensure that the right therapeutic dose will be administered into the patient and for QC of the final product, cell viability testing and cell counting should be conducted prior to lot release based on guidelines by different regulatory authorities worldwide including the FDA, EMA, PMDA, Ministry of Health, Labour and Welfare (MHLW) of Japan and NMPA.^147^, ^151^, ^158^, ^159^ As hiPSC‐derived β cells are cultured as aggregates, a representative sample of aggregates should be taken from each lot and dissociated into single cells with reagents such as TrypLE, for viability and cell‐counting measurement with Trypan blue staining and the use of a haemocytometer. SOPs should be established to ensure consistent cell clump disaggregation for accurate and reproducible reporting of viability and cell number.^160^ Release criteria could be set such that each lot should only be clinically administered if the size of each aggregate is comparable with the diameter of an IEQ. In addition, each aggregate should have a minimum of 70% of viable cells and a consistent cell count.^23^, ^161^

### 5.3 Safety

The safety concerns of hiPSC‐derived cell therapy products largely pertain to its genetic stability in long‐term culture and residual tumorigenic potential. hiPSCs destined for large‐scale cell therapy product manufacturing are likely to undergo prolonged cultivation for continued expansion. However, long‐term cultivation of hiPSCs may result in genetic abnormalities.^162^, ^163^ With the potential for these mutations to confer a growth advantage, it is important to regularly assess the genomic stability of hiPSC‐derived products during the manufacturing process and before product release (Table 3). If aberrations are detected, the final cell product can only be administered when the aberrations are documented to be functionally insignificant.^164^

In addition, as hiPSCs can form teratomas,^165^ residual undifferentiated hiPSCs in hiPSC‐derived β cells pose tumour risks when transplanted. Hence, final hiPSC‐derived β‐cell products should be screened for tumourigenicity, as outlined under FDA, EMA, MHLW and NMPA regulations.^164^, ^166^, ^167^, ^168^ Such tumorigenic studies can be conducted via in vivo transplantation of cells into an immunodeficient mouse for 12–16 weeks. However, the 50% Tumour Producing Dose (TPD50) of different cell lines may vary.^169^ As the number of cells eventually transplanted may have to be scaled down in smaller animal models due to feasibility issues, tumourigenicity potential may not be demonstrated until much later, when larger numbers of hiPSC‐derived β cells are transplanted into humans.

Furthermore, it is not practical to conduct in vivo tumourigenicity studies for batch release due to the long duration required. Therefore, to address the safety concerns of hiPSC‐derived β cells, the use of in vitro assays to examine the propensity of tumour formation for each lot release is recommended by various regulatory bodies.^151^, ^167^, ^170^ For instance, telomerase repeated amplification protocol can be conducted to test for telomerase activity, which should be completely absent in hiPSC‐derived β cells.^171^ With reference to FDA and PMDA, tests focused on detecting residual hiPSCs should also be conducted to properly assess the tumorigenic risk in hiPSC‐derived β cells.^134^ Simple and sensitive methods of detection through quantitative flow cytometry of hiPSC markers as afore‐mentioned for hiPSC characterization can be carried out.^172^

### 5.4 Potency

The choice of potency assay(s) is subjective and dependent on product characteristics. Hence, current global regulations do not dictate a specific type of potency assay to be used for cell therapy products. In accordance with FDA guidelines, the potency assays used should be validated and should be able to directly measure the cell product's activity relevant to its mode of action with accuracy, precision and robustness.^173^ Here, we propose a myriad of in vitro and in vivo assays that can be used to demonstrate the functionality of β cells—secretion of insulin in response to high blood glucose levels to maintain euglycemia.

As the goal of hiPSC‐derived β‐cell therapy is to mitigate the scarcity of cadaveric donor islets for diabetes treatment, the functionality of hiPSC‐derived β cell‐related products should be compared with that of bona fide human islets. The in vitro analysis of a β cell's functionality should include GSIS assays, where the functionality of hiPSC‐derived β cells is demonstrated through an increase in insulin secretion upon glucose challenge.^3^, ^87^, ^114^

In addition, an examination of the calcium signalling involved in insulin secretion will be beneficial to detail the physiological efficacy of β cells in vitro. When glucose levels increase in vivo, uptake of glucose is mediated through glucose transporters, and glucose is then catabolized in the aerobic respiration pathway to generate adenosine triphosphate (ATP).^174^ In response, ATP‐sensitive K^+^ (K_ATP_) channels close and initiate a wave of membrane depolarization, which allows for calcium influx through voltage‐dependent calcium channels and the eventual insulin exocytosis.^174^ To indirectly demonstrate that the hiPSC‐derived β cells are functional, calcium influx assays can be carried out with a calcium indicator dye such as Fluo‐4 AM under glucose challenge.^124^, ^126^

## 6 CURRENT LANDSCAPE, CHALLENGES AND FUTURE PERSPECTIVES

Over the past two decades, protocols to differentiate hPSCs into β cells have been increasingly refined for clinical translation. To date, applications for hPSC‐based diabetes treatment are gradually being materialized, with numerous companies gearing up to bring their technologies into clinical use. Promising clinical trial studies include those from ViaCyte and Vertex Pharmaceuticals (which acquired Semma Therapeutics).

Proof‐of‐concept was recently established in ViaCyte's phase 1/2 clinical trial designed to evaluate the engraftment and efficacy of encapsulated hPSC‐derived pancreatic progenitors (VC‐01) in immunosuppressed T1D patients. These hPSC‐derived pancreatic progenitors were meant to differentiate into glucose‐responsive β cells in vivo. Some subjects with undetectable C‐peptide levels prior to the study were evidenced to have stimulated C‐peptide levels post‐transplantation with VC‐01. Further examination of transplanted sentinel units—meant for withdrawal at various timepoints for characterization purposes, revealed successful engraftment and maturation into insulin‐producing β cells.^175^, ^176^

Semma Therapeutics previously presented that hPSC‐derived islet‐like cells were able to engraft and demonstrate functionality in immunosuppressed non‐human primates over 6 weeks.^177^ Vertex Pharmaceuticals was later awarded the Fast‐Track Designation for VX‐880, whence they commenced the testing of stem cell‐derived islet‐like cells in T1D patients with concomitant immunosuppression in their phase 1/2 clinical trial.^178^ Recently, Vertex published preliminary data announcing a proof‐of‐concept in their first patient, with transplanted hPSC‐derived islet‐like cells demonstrating successful engraftment and glucose responsiveness within 90 days.^179^ Altogether, while preliminary, the viability and functionality of hPSC‐derived β‐like cells underscores its potential in diabetes treatment and furthers the promise of ushering hiPSC‐derived β cells into the clinics.

It should be noted that clinical efforts thus far are mostly geared towards the treatment of T1D patients, where transplantation of hPSC‐derived β cells serve as a direct replacement for the depleted endogenous β cells. Similarly for T2D patients, hPSC‐based cell therapy should first be initiated in those insulin‐requiring T2D patients who no longer have functional β‐cell mass. T2D patients will have varying levels of insulin resistance that may complicate dose‐finding clinical trials since patients will require different therapeutic doses. Additionally, the hurdle of peripheral insulin resistance may not be easily overcome by mere replacement of endogenous β cells. Indeed, rat islets transplanted into streptozocin‐diabetic rats fed with high fat diet were shown to become dysfunctional in a chronically hyperglycaemic and high fat environment that is analogous to T2D.^180^ However, this challenge may be alleviated by simultaneous treatment with T2D drugs and hPSC‐derived β‐cell transplantation. This was demonstrated in a study conducted by Bruin et al.,^181^ where treatment with T2D drugs in combination with hPSC‐derived pancreatic progenitor transplantation led to significantly improved glucose tolerance within 16 weeks in a high fat diet‐induced T2D mouse model. Most notably, treatment of the T2D mouse model with a combination of Sitagliptin and cell therapy resulted in comparable glucose tolerance levels to the low‐fat diet control.^181^ While it is acknowledged that T2D is a chronic disease resulting in a gradual dysfunction of β cells and the longer‐term effects of T2D on this combinatorial strategy are yet to be elucidated, this study demonstrates the potential for β‐cell replacement therapy to slow down, if not reverse, the progression of T2D. As hPSC‐based therapy for T1D becomes a tangible reality, lessons learnt can also be used to make it a reality for eventual T2D treatment.

Despite progression in clinical testing, we still foresee several challenges ahead for widespread adoption of hiPSC‐derived β cells for diabetes treatment. The first pertains to the long‐term preservation and maintenance of hiPSC‐derived β cells. To facilitate the development of an off‐the‐shelf product, hiPSC‐derived β cells must have the capacity to be cryopreserved with no change in cell identity and no loss of viability and functionality post‐thaw.^148^ However, this can be difficult to achieve due to the 3D architecture of hiPSC‐derived β‐cell ‘organoids’. During the freezing process, layers of cells within the organoid structure with different intracellular water potential are exposed to varying temperatures. This culminates in the formation of damaging intracellular ice crystals.^182^, ^183^ Hence, cryopreserved islets/β‐cell organoids generally show a decrease in viability and functionality post‐thaw.^184^ The lack of optimized cryopreservation methods therefore hinders an on‐demand availability of hiPSC‐derived β cells. To that end, research efforts have been geared towards the development of less damaging cryopreservation processes, including dissociation of hiPSC‐derived β cells to single cells for freezing before reaggregation post‐thawing,^185^ encapsulation of β‐cell organoids with cryoprotective hydrogel,^186^ and vitrification.^187^ However, these methods of cryopreservation remain relatively untested, with a lack of comprehensive data on the viability and functionality of these hiPSC‐derived β cells. Hence, with the current state‐of‐the‐art, hiPSC‐derived β cells are likely to be made on demand, with an optimized supply chain procedure in place for near‐immediate transplantation.

The second challenge relates to the immunological rejection that obstructs successful allogenic transplantation. Although immunosuppression drugs can be taken, these medications are life‐long and potentially toxic, which diminishes the benefits of hiPSC‐derived β‐cell therapy.^188^, ^189^ In lieu of that, encapsulation strategies are increasingly being explored to protect the cells from the host immune system and to prevent immune reactions against the encapsulated β cells.^190^, ^191^, ^192^, ^193^ While traditional implantable devices are typically constructed with materials such as silicon and titanium, concerns with nutrient diffusion have pushed researchers to adopt other capsule materials such as alginate and polyethylene glycol.^192^, ^193^ However, as exemplified by ViaCyte's clinical trial, foreign body reactions may still occur, leading to fibrosis around the devices and affecting the viability of the encapsulated cells.^191^ In addition, besides functionality issues, other regulatory concerns regarding biocompatibility, sterility and functionality of these accompanying medical devices will need to be addressed during clinical trials and in product release testing. Depending on the site of transplantation, increasingly stringent regulations may also be imposed. Hence, efforts geared towards the derivation of hypoimmunogenic hiPSC lines for eventual directed differentiation, through deletion of HLA proteins or overexpression of PDL1‐CTLA4Ig molecules which can modulate T‐cell activation, may deliver greater promise for the transplantation of hiPSC‐derived β cells.^194^, ^195^

In addition, given the novelty and complexity of cell therapy products for clinical treatment, regulatory authorities may also be hesitant to define the potency testing(s) required and implement rules for QC checkpoints. Here, we have attempted to outline suggested potency and QC testing based on current standards. However, these proposed tests will still be subjected to regulatory oversight. Without additional clear guidelines, bench‐to‐clinic translation may be hindered. To facilitate the commercialisation of regenerative medicine products, collaborations with expert panels to establish guidelines for preclinical and clinical testing will be helpful. Harmonization of standards will also be useful to facilitate a wider adoption of hiPSC‐based cell therapy.

Last but not least, for manufacturers focused on cell therapy, the considerations for personnel involved may be different from that of an academic setting, where diverse interdisciplinary collaboration will be required early in the development of the product (Figure 1). For instance, the expertise of process engineers will be needed for developing scale‐up manufacturing and optimization of encapsulation devices whereas stem cell biologists will be needed to scrutinize cell culture reagents and growth factors used in the hiPSC culture, expansion, and differentiation processes. In addition, individuals with the legal and regulatory expertise will also be required to facilitate regulatory compliance with the jurisdiction of various countries. If encapsulation devices are to be incorporated into the final product, under EU regulations, a person responsible for regulatory compliance will also need to be appointed.^196^ Close partnership with clinicians and hospitals will certainly need to be established for widespread data collection on patient safety to conduct appropriate risk management and monitoring. Hence, other than a regulatory framework that needs to be established within the company, careful planning of the required various job roles should be carried out as early as possible.

In conclusion, it is now possible to generate clinically compliant hPSC‐derived β cells with higher yield and better functionality in vitro. Further harmonization of the regulation of hPSC‐based cell products and even optimisation of cryopreservation methods should be made to pave the way for its upcoming clinical translation and commercialisation. For industries focused on hPSC‐based cell therapy, the establishment of a complete team that can address all the considerations listed above will be necessary in order to progress towards clinical trials. By surmounting these obstacles, it is highly probable that hiPSC‐derived β cells have a chance of being a curative treatment for diabetes patients.

## CONFLICTS OF INTEREST

Adrian Kee Keong Teo is a co‐founder of BetaLife Pte Ltd. Other authors declare no conflicts of interest.

## AUTHOR CONTRIBUTIONS

*Conceptualization*: Adrian Kee Keong Teo. *Investigation*: Lay Shuen Tan and Juin Ting Chen. *Writing – original draft*: Lay Shuen Tan and Juin Ting Chen. *Writing – review and editing*: Lay Shuen Tan, Juin Ting Chen, Lillian Yuxian Lim, and Adrian Kee Keong Teo. *Funding acquisition*: Adrian Kee Keong Teo.

## Data Availability

Data sharing is not applicable to this article as no new data were created or analyzed in this study.
